# Prevalence, risk factors and consequences of intra-team conflicts in the ICU

**DOI:** 10.1186/cc13211

**Published:** 2014-03-17

**Authors:** G Burghi, F Verga, M Acevedo, V Martinez, V Carrasco, C Quiroga, G Pittini, G Fariña, M Godino, S Mareque, P Alzugaray, H Bagnulo, E Azoulay

**Affiliations:** 1 Hospital Maciel, Montevideo, Uruguay; 2 COMERO, Rocha, Uruguay; 3 Sanatorio Americano, Montevideo, Uruguay; 4 Hospital Español, Montevideo, Uruguay; 5 CAAMEPA, Pando, Uruguay; 6 Casa de Galicia, Montevideo, Uruguay; 7 CAMS, Mercedes, Uruguay; 8 Hopital Saint Louis, Paris, France

## Introduction

The ICU is a stressful place with several sources of conflicts. Among ICU workers, conflicts lead to burnout, loss of psychological well-being, and deterioration of the work performance. Intra-team conflicts (conflicts that occur among members of the intensive care team) are one of the most frequent types of conflicts in the ICU. The objectives of this study were to determine the prevalence, risk factors and consequences associated with intra-team conflicts in the ICU.

## Methods

A survey was conducted in 12 Uruguayan adult ICUs in 2009. ICUs and clinician's characteristics were assessed for their association with the prevalence of conflicts. All factors associated with conflicts were introduced into a multivariable model.

## Results

A total of 384 questionnaires were collected, 74.5% (*n *= 286) were nurses and 23.2% (*n *= 89) were intensivists. From the 384 forms, 45.8% perceived at least one conflict in the past year. Conflicts were perceived more frequently by intensivists (61.8%) than nurses (40.9%) (*P *= 0.001). A worse relationship with intensivists (OR 1.3; 95% CI 1.09 to 1.59; *P *= 0.004) is independently associated with conflicts. However, lower grade of irritability (OR 0.8, 95% CI 0.73 to 0.97; *P *= 0.01) and a higher position in the iCu (OR 0.57 95% CI 0.36 to 0.92; *P *= 0.02) are factors independently associated with a lower incidence of intra-team conflicts. Interestingly, this study confirms that workers with at least one conflict had more frequently: libido disturbances (56% vs. 40%; *P *= 0.002), eating problems (52% vs. 40%; *P *= 0.02), as well as the wish to leave the ICU (53% vs. 39%; *P *= 0.005).

## Conclusion

The prevalence of conflicts is very high among ICU workers in Uruguay. We have identified different risk factors associated with the development of conflicts. These results confirm previous findings and highlights that strategies to decrease intra-team conflicts in the ICU are urgently warranted.

